# Items

**Published:** 1874-09

**Authors:** 


### Items.

The Board of Commissioners of Public Charities and Corrections have determined to make the medical board of Bellevue Hospital, consist of sixteen instead of nineteen members as originally determined, and have therefore made the following additions to the staff. From Bellevue College, Drs. F. H. Hamilton and Janeway; University, Drs. W. H. Thompson and Erskine Mason; College of Physicians and Surgeons, Drs. Francis Delafield and

Thomas M. Markoe; at large Drs. J. W. S. Gouley and Gouverneur M. Smith.——Dr. Wm. H. Hammond has sued the New York *Medical Record* for libel alleged to have been contained in an editorial in that Journal for Aug., 1st, 1874, upon Hydrophobia.——It is expected that the first volume of the Cyclopædia of the Practice of Medicine, will be issued during October, it is now running through the press, and specimen pages can be obtained on application to Messrs Wm. Wood & Co.——Dr. Geo. M. Beard, No. 53 W. Thirty-third St., New York, is seeking to obtain information concerning "Hay Fever" or "Autumnal Catarrh, and especially does he desire information upon those facts, which seem to indicate the dependence of the disease upon the Nervous System. He has prepared a circular setting forth a number of questions, which he will furnish upon application.

